# Fast 4D On-the-Fly Tomography for Observation of Advanced Pore Morphology (APM) Foam Elements Subjected to Compressive Loading

**DOI:** 10.3390/ma14237256

**Published:** 2021-11-27

**Authors:** Michal Vopalensky, Petr Koudelka, Jan Sleichrt, Ivana Kumpova, Matej Borovinsek, Matej Vesenjak, Daniel Kytyr

**Affiliations:** 1 Czech Academy of Sciences, Institute of Theoretical and Applied Mechanics, Prosecka 809/76, 190 00 Prague, Czech Republic; koudelkap@itam.cas.cz (P.K.); sleichrt@itam.cas.cz (J.S.); kumpova@itam.cas.cz (I.K.); kytyr@itam.cas.cz (D.K.); 2 Faculty of Mechanical Engineering, University of Maribor, Smetanova Ulica 17, 2000 Maribor, Slovenia; matej.borovinsek@um.si (M.B.); matej.vesenjak@um.si (M.V.)

**Keywords:** 4D CT, microcomputed tomography, on-the-fly tomography, image quality, advanced pore morphology (APM) foam, in-situ mechanical testing, compressive loading

## Abstract

Observation of dynamic testing by means of X-ray computed tomography (CT) and in-situ loading devices has proven its importance in material analysis already, yielding detailed 3D information on the internal structure of the object of interest and its changes during the experiment. However, the acquisition of the tomographic projections is, in general, a time-consuming task. The standard method for such experiments is the time-lapse CT, where the loading is suspended for the CT scan. On the other hand, modern X-ray tubes and detectors allow for shorter exposure times with an acceptable image quality. Consequently, the experiment can be designed in a way so that the mechanical test is running continuously, as well as the rotational platform, and the radiographic projections are taken one after another in a fast, free-running mode. Performing this so-called on-the-fly CT, the time for the experiment can be reduced substantially, compared to the time-lapse CT. In this paper, the advanced pore morphology (APM) foam elements were used as the test objects for in-situ X-ray microtomography experiments, during which series of CT scans were acquired, each with the duration of 12 s. The contrast-to-noise ratio and the full-width-half-maximum parameters are used for the quality assessment of the resultant 3D models. A comparison to the 3D models obtained by time-lapse CT is provided.

## 1. Introduction

Recent advancements in radiation detector technology has allowed the decrease of exposure times in scintillation detectors to the order of tens of milliseconds with an acceptable image quality even under common laboratory conditions. When computed tomography (CT) is used to create the virtual model of a static object, the scan duration is usually not a limiting factor. However, CT has been gaining increasing attention as a powerful method for investigating dynamic processes, such as loading experiments in mechanical testing [1]. In these applications, a series of subsequent tomographic scans is acquired during the course of the mechanical experiment. The resulting 3D virtual models can be set together to form a 3D movie of the process. Hence, the time is added as the fourth dimension to the 3D tomography, therefore the term 4D CT. The more scans are performed in the given time frame, the higher is the temporal resolution of the result, improving the possibilities of the analyses of changes in the reconstructed volumes between particular scans. However, making extensive sets of tomographic scans is a very time-demanding task. The best results from the viewpoint of imaging quality, which directly influences the resolution in the resultant virtual model, are achieved when the scanned object is static throughout the CT scan. In certain experiments and with appropriate instrumentation, the dynamic process can be observed in discrete load-steps (i.e., suspended for the tomographic scan and continued with the next step between two subsequent scans). This method is often referred to as a time-lapse 4D tomography or intermittent 4D tomography [2,3,4,5]. Nevertheless, such an intermittent loading procedure of the dynamic process in question can be hard to realize in practice. Even then, it is desirable to keep the scanning time as short as possible. There is yet another possibility for acquiring the series of tomographic scans: the process runs continuously without any interruptions, simultaneously with continuous CT scans, in the so-called on-the-fly or real-time imaging procedure [6]. In that case, the scanning must be fast enough so that the observed process can be considered as “quasi-static” during one CT scan (one full revolution of the investigated object). The required acquisition frame rate is then determined by the number of projections per revolution, the magnification, and the speed of the process itself. Additionally, during the standard CT, the rotational platform stops in the correct angular position to avoid blurred images caused by the object movement. However, accelerating and decelerating the rotational platform and settling in the correct angular position with a high precision takes additional time. Fortunately, it may be possible to neglect the object movement during the exposure when sufficiently short exposure times are used. Consequently, if the rotational speed of the platform can be considered constant and stable, the acquisition of the tomographic series can be done without suspending neither the platform nor the process.

One of the recent advancements in the field of porous materials for structural applications is represented by the development of advanced pore morphology (APM) foam elements, quasi-spherical elements with a closed-cell foam core encapsulated by a solid outer layer [7,8]. So far, a wide range of their applications has been proposed, including deformation energy absorption [9], vibration damping [10], and applications comprising multi-layered composite structures [11,12]. Furthermore, one of the most significant advantages of the APM foam elements is the ability to be easily combined with different types of filling materials resulting in multi-phase hierarchical materials. Here, the APM elements can be used to produce unbonded or polymer-bonded hybrid porous structures [13,14], or syntactic APM foam with polymer-based matrix when the void space between the APM elements is filled with a filling material [15,16].

Similar to other closed-cell foam-based materials, the mechanical properties of APM elements are dependent upon topological and morphological characteristics, such as pore size distribution and internal porosity distribution. Numerous teams have studied the mechanical properties of APM elements. The approach adopted by the researchers was predominantly based on a combination of advanced computer simulations coupled with experimental testing [14,17,18,19]. Volumetric characterization of their internal structure was established from X-ray µCT using both the intact and deformed APM specimens at a micrometric resolution [7,8].

A common shortcoming of many studies concerning the simulation-aided investigation of APM foam mechanical characteristics is the concentration on either the effective deformation properties or the morphological changes in the internal structure. However, such an approach does not provide information on how the deformation of internal structure influences the overall mechanical response of an APM foam element. A possible solution is the use of advanced radiographical imaging methods to reveal the deformation processes within the microstructure of the specimen during a loading procedure [3,20,21,22]. Herein, the time-resolved 4D µCT experiments based on in-situ loading of a specimen in the X-ray scanner that is used for its simultaneous imaging enables capturing the deforming microstructure of the observed APM sample. This approach allows for a detailed investigation of the dominant factors influencing the mechanical properties of the sample.

According to our previous study [23], such a 4D µCT experiment with APM elements can be successfully performed using a loading procedure composed of discrete loading steps. However, given the pore size distribution, dimensions of the cell walls, and overall dimensions of the samples, the relaxation of the sample after each loading step may significantly extend the duration of the experiment and affect the acquired mechanical data. Due to the release of elastic energy accumulated in the specimen and the loading device during the loading stage, the resulting specimen displacements during the relaxation process may be comparable with the resolution of the CT scanner, reducing the quality of the resulting reconstructed 3D volume. From the mechanical viewpoint, the repeated load changes between the individual load steps may influence the deformation response as the load often tends to concentrate in the weakest elements of the microstructure leading to a different global response (i.e., formation of shear planes).

Therefore, it is reasonable to consider the on-the-fly CT loading, as described above: the sample is continuously loaded and simultaneously scanned during the experiment without suspension. After the reconstruction, this results in 3D models that capture in detail the sample deformation [24,25]. As we have shown in our previous paper aimed at on-the-fly imaging of a deforming tissue scaffold, the limiting factor here is the achievable strain-rate which should guarantee a sub-voxel deformation of the sample during one CT scan (i.e., one revolution of the sample in the CT setup) not to sacrifice the quality of the reconstructed 3D volume. Thus, it is obvious that a decrease in the CT scan duration may significantly extend the allowable strain-rate range of the on-the-fly experiments, which is a fundamental factor in the field of deformation energy absorption applications.

**Figure 1:**
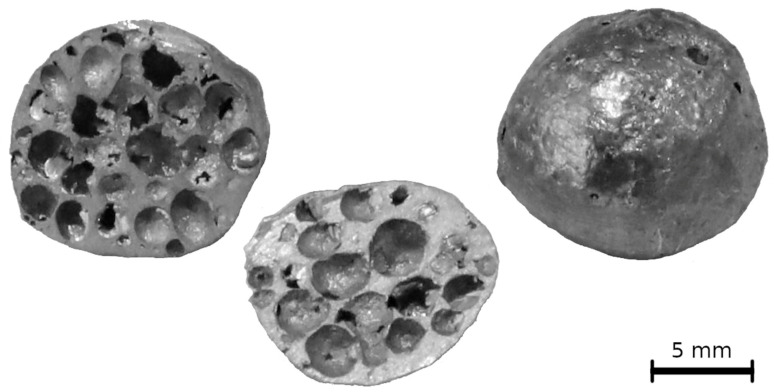
APM foam elements used in the experiment.

In this study, a commercially available scintillation detector with a resolution of 768 px × 972 px was used to capture the deformation process of the APM foam elements (Figure 1), with the resultant voxel size of 15.2 µm. It provided the acquisition time of 15 ms, i.e., the acquisition rate of over 60 fps (frames per second). With approximately 800 projections per revolution, this means that one CT scan was made within 12 s. Every second revolution was used for saving the data. Thus, the real-time resolution of the 3D sequence was 24 s. Such a rate is standardly realizable in synchrotron facilities, but it is very fast for the current laboratory X-ray setups. The resultant 3D models have been compared in terms of the imaging quality to the tomographic models made out of CT scans of static APM elements and tomographic models made using double resolution scans.

## 2. Instrumentation

### 2.1. Loading Device

**Figure 2:**
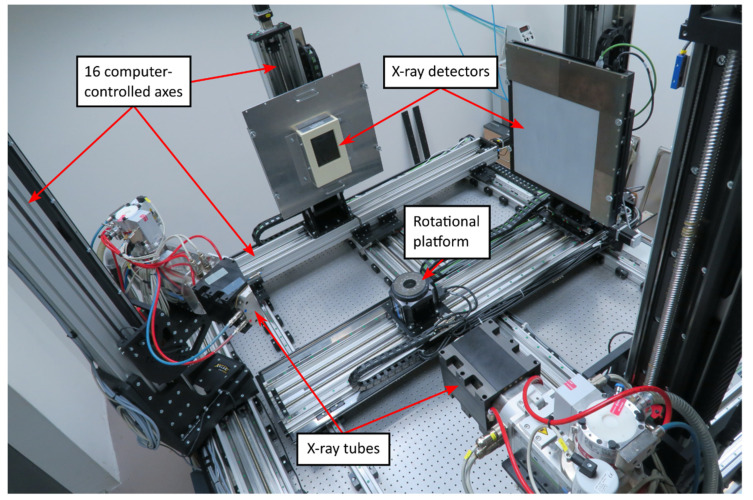
TORATOM scanner.

**Figure 3:**
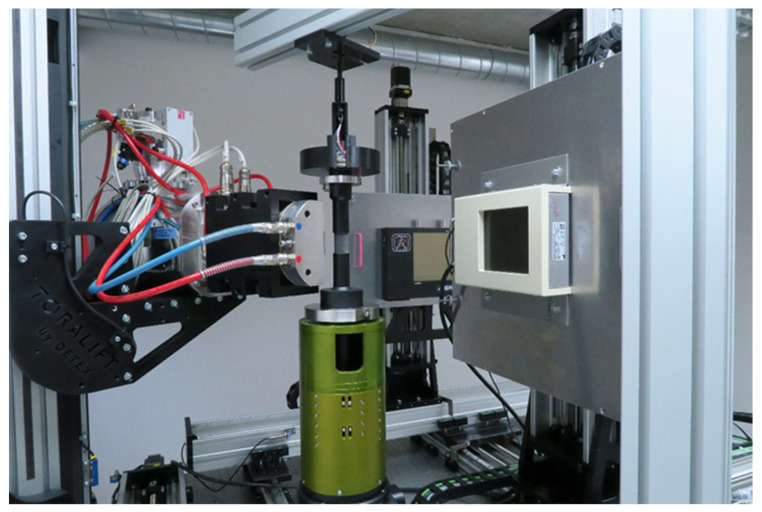
Experimental setup comprising the in-situ loading device mounted into TORATOM.

The in-situ compressive experiments were performed using a modified in-house designed table-top loading device allowing on-the-fly 4D µCT experiments [4]. The modification consisted in using a 20 mm diameter 3-ply carbon fiber tube to allow the shortest possible distance of the object from the X-ray source to achieve the highest possible geometrical magnification. A 1000 N load-cell was used for force measurements. The device was integrated with the high-precision rotary stage of the in-house developed twinned orthogonal adjustable tomograph (TORATOM), depicted in Figure 2, using slip rings to allow its free rotation during the loading procedure. Figure 3 shows in detail the configuration of the measurement setup with the loading device accommodating the APM sample inserted into the scanner. The high precision of the force measurement was achieved by correction for the overall stiffness of the loading device and by assessing its long-term stability in both the measured force and loading platen position.

### 2.2. Microcomputed Tomography

A TORATOM device [26] was used for the imaging of the experiment. TORATOM is a patented, in-house made complex tomograph with two orthogonal imaging axes, i.e., with two X-ray sources and two detector stands. The high-precision rotational platform Aerotech APR150DR-135 [27] is shared between both axes. The vertical and horizontal positions of the tubes and the detectors, as well as the horizontal position of the rotational platform, is adjustable in a broad range, making TORATOM an outstandingly versatile device. There are in total 16 computer-controlled axes; the mechanical resolution step of the crucial ones (source-to-detector distance, detector height) being 1 µm. The maximum source-to-detector distance (FDD) can be set up to approximately 1250 mm in both imaging axes. There is the possibility of simultaneous imaging in both axes. In that case, the minimum FDD is 530 mm. In the case of a single-axis imaging mode, the lowest FDD is below 300 mm, limited by the size of the rotational platform body carrying the scanned object.

The loading device was put directly onto the rotational platform (see Figure 3). For the experiment, 240 kV reflection type tube [28] was used as the X-ray source. Dexela 1512T detector [29] with a GOS scintillator operating in the Low Well mode was employed for image acquisition. The full resolution of the detector is 1944 px × 1536 px. In full resolution, the minimum exposure time is approximately 40 ms. Pixel binning 2 × 2 leads to the resolution of 972 px × 768 px, but the minimum exposure time reduces to 15 ms. The FDD was set as short as possible to maximize the incident beam intensity at the detector plane, which is proportional to the reciprocal value of the square of FDD. The shortest FDD is limited by the requirement that the active detector area fits entirely into the X-ray cone. Considering the dimensions of the detector active area of 145.4 × 114.9 mm^2^ and the cone beam aperture of 30°, the resultant minimum FDD is 345 mm. Taking into account the diameter of the non-deformed APM sphere being approximately 10 mm and leaving enough space in around the sphere in the projection, it was decided that the magnification would be 9.85 by setting the distance between the radiation source and the rotation axis (focus-to-object distance, FOD) to 35 mm. Consequently, the diameter of the projected non-deformed APM sphere covers approximately 85 % of the detector height.

## 3. Experimental

In total, three APM foam elements made of AlSi7 alloy were used in the experiment. The samples were fabricated by powder metallurgy process (heating the compacted precursor consisting of AlSi7 alloy and TiH2 foaming agent) at Fraunhofer IFAM Bremen, Germany, with an expected nominal diameter of 10 mm and porosity of approximately 75 % [8,14,23].

As can be seen in Figure 1, the samples were of quasi-spherical shape with an outer diameter of 10.6 ± 0.5 mm, a meridian diameter of 10.0 ± 0.3 mm. The weight of the samples was 378.7 ± 2.2 mg. The uni-axial compressive tests were controlled using an in-house developed control software. The loading force was measured using a load-cell signal with 0.1 N precision and the position was established from an encoder signal with 0.25 µm resolution. A sensor readout rate was of 100 Hz. The device stiffness correction curve was derived from calibration measurements consisting of plate-to-plate calibration tests resulting in 180 µm/kN of calculated device stiffness.

At first, CT scans with 2400 projections per revolution (Mode A) and 800 projections per revolution (Mode B) with the table suspended in the correct angle during the image acquisition and without any loading applied during the rotation were performed. Such a procedure corresponds to the scanning in the time-lapse mode, as performed in [23]. The resolution of the detector was 1944 px × 1536 px (width × height) and the exposure time was set to 90 ms. In the second step, the second APM foam element was scanned in the on-the-fly mode, but without loading, with 2400 projections per revolution (Mode C) and 800 projections per revolution (Mode D). Then, the on-the-fly scans with 800 projections per revolution and the loading rate of 2.5 µm/s (Mode E) was realized. Since the APM element was deformed during this experiment, the third APM element was taken and compressed at the rate of 5 µm/s (Mode F). Obviously, there is a certain angular movement during each exposure in the on-the-fly modes (Mode C–F).

In Modes C–F, the resolution was reduced to 972 px × 768 px due to 2 × 2 binning and the exposure time was set to 15 ms. In all modes, the tube acceleration voltage was set to 90 kV. This value was chosen with regard to the intensity ratios at the detector behind the object and around the object to maximize the contrast in the images [30]. The target power was of 30 W in Mode A and B and the detector was operated in the less-sensitive High Well acquisition mode. In Mode C–F, the detector acquisition mode was set to the high-sensitive Low Well and the target power was lowered to 25 W not to saturate the detector. In all modes, the magnification was kept at approximately 9.86, yielding the pixel size of7.6 µm in full-resolution (Mode A, B) and 15.2 µm with 2 × 2 binning (Mode C–F).

Making 800 projection images during one rotation (i.e., one CT scan) was found to be a reasonable compromise between the reconstructed model quality and the duration of the on-the-fly procedure. With the acquisition time of 15 ms per projection, obtaining 800 projections took 12 s. The total number of images taken in the experiment was technically limited to 33,000. With 800 projections per one CT scan, 41 full CT scans could be acquired. The rotational platform was rotating at a constant speed of 360°/12 s, or 30°/s during the entire experiment. For the successful reconstruction of an object scanned in the on-the-fly mode, it is necessary to ensure that the exposure time is stable and identical for each projection to guarantee a constant angular shift in each projection. Therefore, any delaying operations had to be avoided during the image acquisition. In the time-lapse CT modes (Mode A, B), the individual projections were flat-field corrected after the acquisition and immediately saved to the disk causing an increase of the real time for one projection (the exposure time was of 90 ms, but the time needed to complete one projection was more than 500 ms). In the on-the-fly modes (Mode C–F), however, the acquired uncorrected images from one complete revolution were saved in a batch and the flat-field correction was performed on all datasets during post-processing. In this way, the real time for one projection was not increased by additional operation and it stayed equal to the exposure time of 15 ms. The on-the-fly scanning procedure was as follows: The Dexela detector acquired 800 images, then the acquired images were saved to the disk. However, the rotational platform kept moving during saving and the acquisition of the next 800 images started when another revolution was completed. Hence, only every second rotation of the object was recorded.

The dark-field image for on-the-fly modes was acquired as a median of 800 exposures without illumination; the open beam image as a median of 800 exposures on the illuminated detector without the object. The bad pixel map was generated by evaluating the difference in pixel values in the dark image and open beam image. The dark image, open beam image and bad pixel map image were used for the post-processing of all acquired datasets. The datasets were then reconstructed sequentially in the VG Studio Max 3.4 software (Volume Graphics GmbH, Heidelberg, Germany), using the FDK filtered back-projection algorithm [31].

The intended deformation during the on-the-fly experiment was 24% in the case of the first two APM spheres. Considering the diameter of the sphere being approximately 10 mm, the corresponding movement of the loading plate was 2.4 mm. Since only every second rotation is recorded, 984 s of the process are recorded during 41 full scans. The first rotation is made without loading, so that the required deformation must happen during 960 s, leading to the loading rate of 2.4 mm/960 s = 2.5 µm/s. During one scan lasting 12 s, the loading plate moves by 12 × 2.5 µm = 30 µm, which corresponds to almost twice the pixel size. Hence, the mechanical change during one scan is not lower than the pixel size, but as seen from the results, the 2 px change is still acceptable from the point of view of image sharpness. To investigate the effect of even larger mechanical deformation during one scan, the third APM sample was deformed at the double loading rate of 5 µm/s. This led to the mechanical deformation of roughly 4 px during one scan and the total deformation of 48%. The mechanical loading continued even after the on-the-fly scanning in Mode E and Mode F until the displacement of 6 mm was reached. Settings of all the mode-specific scanning parameters are summarized in Table 1.

## 4. Results

### 4.1. In-Situ Compressive Test Results

**Figure 4:**
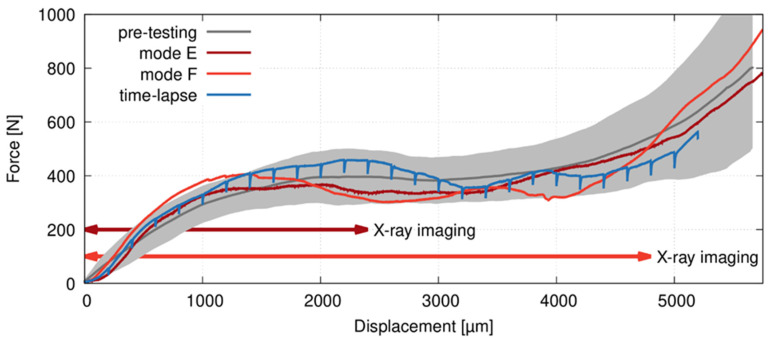
Force-displacement diagrams of the preliminary tests on 6 samples (gray line—mean values, gray area—mean value ± standard deviation); the two samples subjected to the on-the-fly CT scanning; and a sample subjected to the time-lapse measurement for comparison to show the force decreases caused by material relaxation during every CT scan.

To assess the representative mechanical response of the APM foam elements in compression and its variability, six specimens were subjected to the loading procedure without X-ray imaging. The mechanical data acquired during preliminary compressive tests, time-lapse CT with intermittent loading and on-the-fly CT imaging are depicted in a force-displacement diagram in Figure 4. All curves were corrected considering the stiffness of the loading device and are in a good agreement with the previously published results [18].

It can be seen that the deformation response of the APM samples in all experiments followed similar shape typical for cellular materials, where an increase of force up to the local maximum is followed by the plateau region. Then, the force progressively increases as the transition to the densification region is reached at the support displacement of 5 mm, corresponding to the total strain of approximately 50%. The graphs also demonstrate the difference in response of the specimens subjected to the time-lapse CT, where the loading is intermittent and the force drops of 10–15% occur due to the release of elastic energy stored in the specimen and the loading device itself causing the measured relaxation effect [23]. Herein, at least 2 min delay between the stop of load increase and the start of a tomographic scan is necessary to deal with the relaxation effect resulting in blurred reconstructed 3D volumes.

### 4.2. Microcomputed Tomography Results

#### 4.2.1. Evaluated Parameters

**Figure 5:**
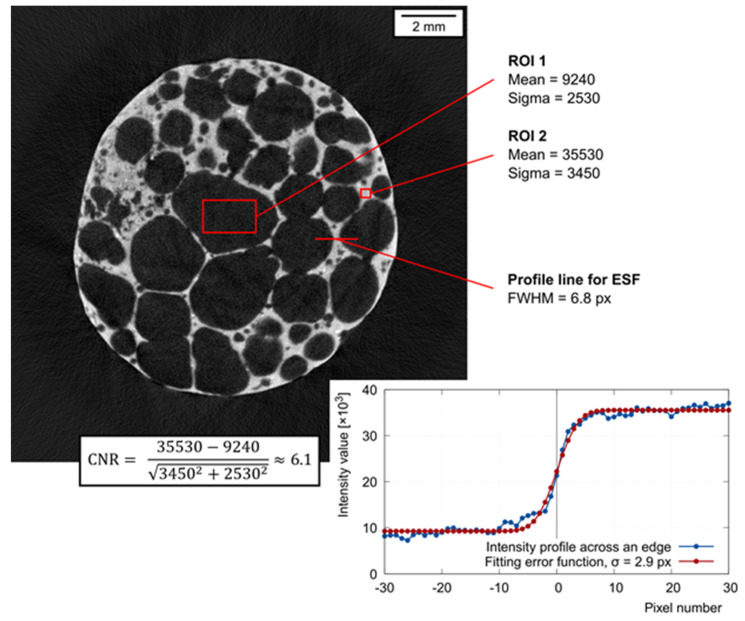
Transversal section of the tomographic model of the APM foam element. ROI1, ROI2—dark and bright area for the calculation of CNR; intensity profile across the edge is fitted with an error function and the FWHM is estimated.

To assess the usability of the fast on-the-fly scanning procedure described in this paper, it is important to quantify the image performance and compare it to the conventional CT with a static object and suspended rotational platform. From this point of view, a crucial parameter is the real resolution of the reconstructed volumes. The resolution is influenced by the pixel size, the noise, and the sharpness of the image. Therefore, the contrast-to-noise ratio (CNR) and the full-width-half-maximum (FWHM) of the line spread function (LSF) are reasonable indicators to be evaluated and compared. Let us consider the tomographic slice of the APM sphere in Figure 5. Let us define two regions of interests (ROI), one in the bright area of the image (ROI2 in Figure 5) and one in the dark area of the image (ROI1 in Figure 5). The contrast *c* can be considered as the difference of mean intensities in ROI1 and ROI2. In each ROI, the noise root-mean-square (RMS) value *σ*_1,2_ is estimated as the standard deviation of the pixel intensities. Then, the CNR can be calculated as

(1) $$CNR=\frac{c}{\sqrt{\sigma_{1}^{2}+\sigma_{2}^{2}}}$$

The intensity profile across a sharp edge in the image is called the edge spread function (ESF) and its derivative is the line spread function LSF, having a bell-shape. The width of LSF in 50% of its height is the FWHM value. The lower FWHM, the sharper the investigated edge and the better the image resolution. The ESF extracted from an actual radiographical image or a tomographic slice can be significantly affected by noise, which prevents a simple calculation of the LSF as the discrete derivative of ESF, since the noise becomes even more pronounced after differentiation, masking the bell shape of the LSF completely. To overcome this difficulty, we can make a reasonable assumption that the LSF is a Gaussian function. Two basic parameters of a Gaussian function are the mean *µ* and the standard deviation *σ*. It can be shown [32] that the FWHM of a general Gaussian function can be expressed as

(2) $$FWHM=2\cdot \sqrt{2\cdot \ln\left(2\right)}\cdot \sigma \approx 2.3548\cdot \sigma$$

As mentioned, fitting of the LSF generated from actual data is difficult due to the noise. However, if a Gaussian function is used to approximate the LSF, then the ESF must be approximable with the integral of the Gaussian function, which is the error function erf. Fitting of the ESF with the appropriate erf is a relatively simple task. Parameter σ can be derived from the fitting erf, and subsequently, the FWHM can be calculated. In Figure 5, the FWHM is approximately 6.8 pixels. The pixel size in Figure 5 corresponds to 15.2 µm, giving the FWHM ≈ 103 µm.

#### 4.2.2. Comparison of Imaging Performance in Mode A–Mode F

**Figure 6:**
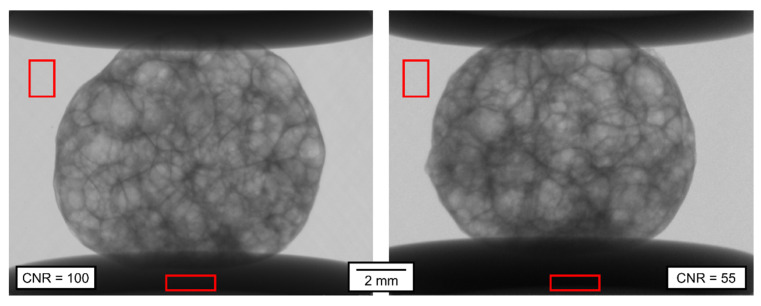
Comparison of the radiographic projections of the APM foam elements. **Left**: Mode B; **Right**: Mode D. ROIs for CNR evaluation indicated in red.

Figure 6 shows the comparison of a single projection made in Mode B (left) and in Mode D (right). While in Mode B, the APM foam element was completely static during the exposure, it was being moved by 0.45° during the 15 ms lasting exposure in Mode D. The lower structural resolution in Mode D projection compared to Mode B projection is notable visually, caused by lower resolution of the projection image itself and by the movement of the object during the exposure in Mode D. Additionally, because of the much shorter exposure time, the Mode D on-the-fly projection exhibits more noise than the Mode B static projection. This fact is again notable visually, but to quantify it, the CNR was evaluated considering the bright region in the background and the dark region in the shadow of the loading plate as the evaluation ROIs. The CNR of the static, 90 ms exposure time projection, is approximately 100, compared to approximately 55 in the case of the on-the-fly projection with 15 ms exposure time. The FWHM was not evaluated in the projections from Figure 6 for the lack of suitable sharp edges.

**Figure 7:**
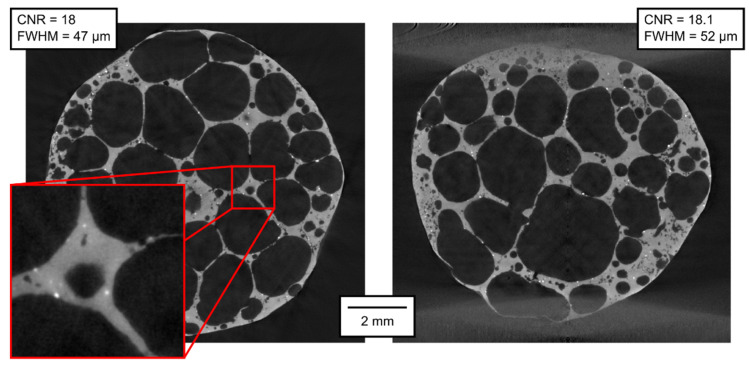
Transversal and lateral central section of the 3D reconstructed volume, Mode A.

**Figure 8:**
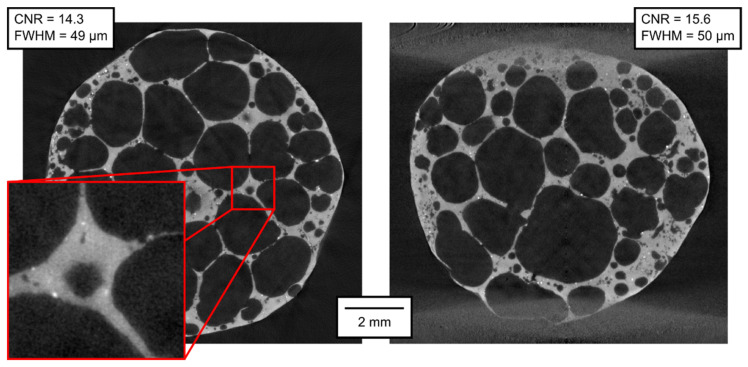
Transversal and lateral central section of the 3D reconstructed volume, Mode B.

**Figure 9:**
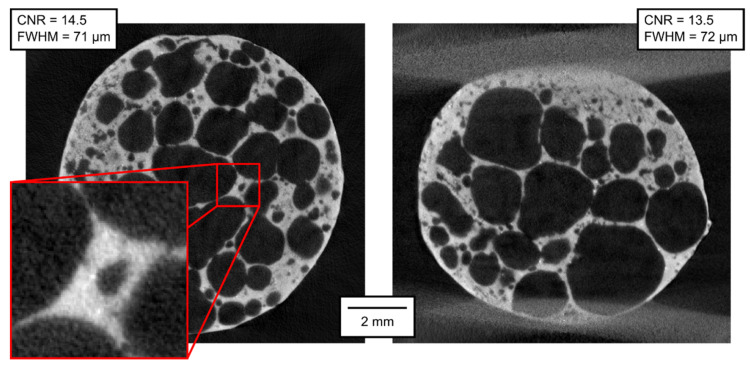
Transversal and lateral central section of the 3D reconstructed volume, Mode C.

**Figure 10:**
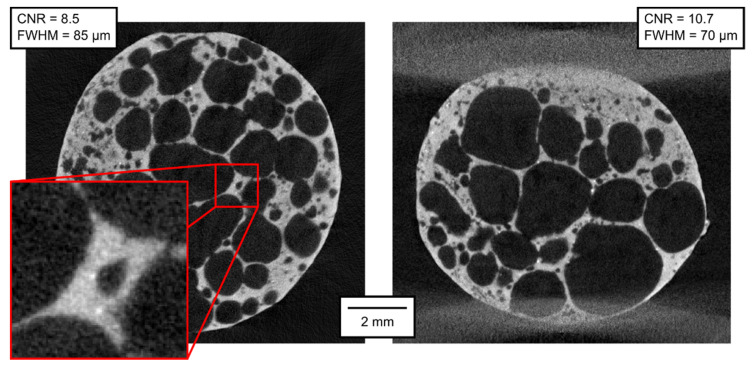
Transversal and lateral central section of the 3D reconstructed volume, Mode D.

**Figure 11:**
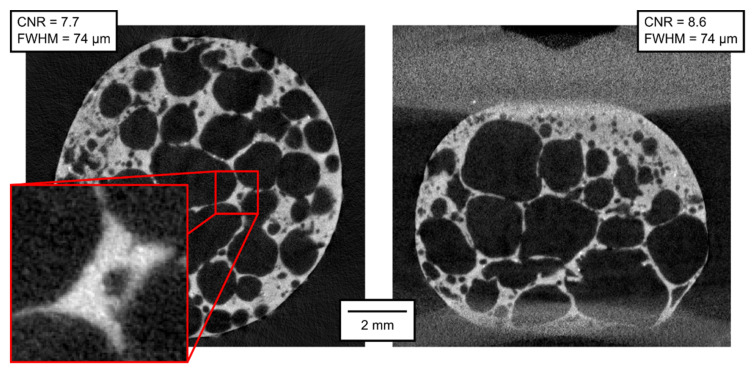
Transversal and lateral central section of the 3D reconstructed volume, Mode E.

**Figure 12:**
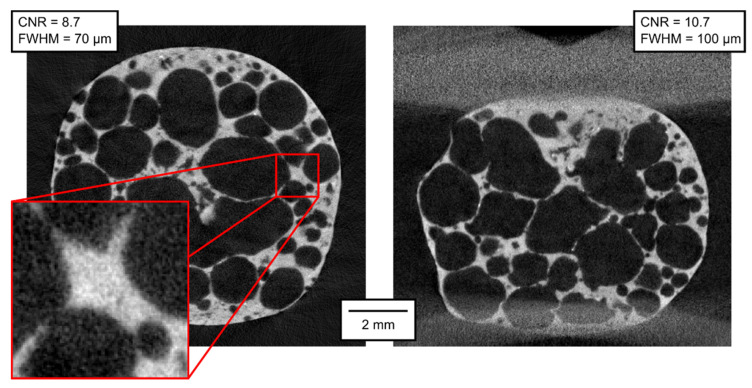
Transversal and lateral central section of the 3D reconstructed volume, Mode F.

The qualitative parameters of the reconstructed 3D models are of much higher interest than the parameters of the projections themselves. In Figure 7, Figure 8, Figure 9, Figure 10, Figure 11 and Figure 12, transversal and lateral sections are shown from the reconstructions of the experiments made in Mode A–F. Obviously, the FHWM parameter is significantly dependent on the chosen edge. Therefore, the indicated FWHM was calculated as the average of five edges in each image. In the case of lateral slices, the evaluated intensity profile lines were always vertical, i.e., in the direction of the loading plate displacement. The FWHM was calculated first in pixels and then converted to the actual length units. The identified parameters of CNR and FWHM are summarized in Table 2. The images presented for Mode E and F correspond to the 12% deformation (i.e., 20th CT in Mode E, 10th CT in Mode F).

## 5. Discussion

As expected, the best imaging quality of the 3D volumes was reached in Mode A, where static APM foam element was scanned with 2400 angular steps per 360°, with the detector resolution of 1944 px × 1536 px. In this case, the CNR was approximately 18 and the FWHM approximately 50 µm. The scanning parameters in Mode B are identical to those in Mode A, with the exception of the number of steps per rotation, which was lowered to 800. A lower number of projections leads to a visually observable increase of noise in the reconstruction; the CNR fell to approximately 15 (see Table 2). However, the FWHM was not affected.

Modes C to F are all made with six times shorter exposure time and with the pixel binning 2 × 2, reducing the resolution to 972 px × 768 px. The reduction in imaging quality compared to Mode A and B is visually obvious. Considering the exposure time and the binning as important factors (although not the single ones) influencing the noise in the reconstructed volumes, we can conclude that six times shorter exposure time leads to increased noise level by the square root of six, but at the same time, as the value in each binned pixel is derived from the values in four adjacent pixels, the noise is reduced by a factor of 2 (the square root of four). Hence, a roughly estimated expectation of the CNR reduction when the rest of the conditions is kept identical is 2/√6 ≈ 0.82. Mode C represents 2400 projections per 360° with the on-the-fly acquisition procedure without applied deformation. The CNR in Mode C is approximately 14, corresponding very well to the expected value derived from Mode A (18 × 0.82 = 14.76). The FWHM is approximately 70 µm, i.e., worse than in Modes A and B, but still not at the half value, which could have been expected considering the half resolution. Mode D, acquisition of 800 projections per 360° without applied deformation, exhibits further fall of CNR, being of 8.5 in the transversal and of 10.7 in the lateral section (even better than calculated from Mode B using the reduction factor of 0.82). However, the FWHM in Mode D is 85 µm in the transversal section and 70 µm in the lateral section, i.e., comparable to Mode C. The CNR stays at similar levels even in Mode E (800 projections per 360°, deformation corresponding to 2 px per rotation) and Mode F (800 projections per 360°, deformation corresponding to 4 px per rotation). The FWHM also stays at the values around 75 µm, except the vertical FWHM in the lateral section of Mode F, being 100 µm. In this case, the influence of the loading during the tomography might begin to play a role in the deterioration of the image sharpness.

**Figure 13:**
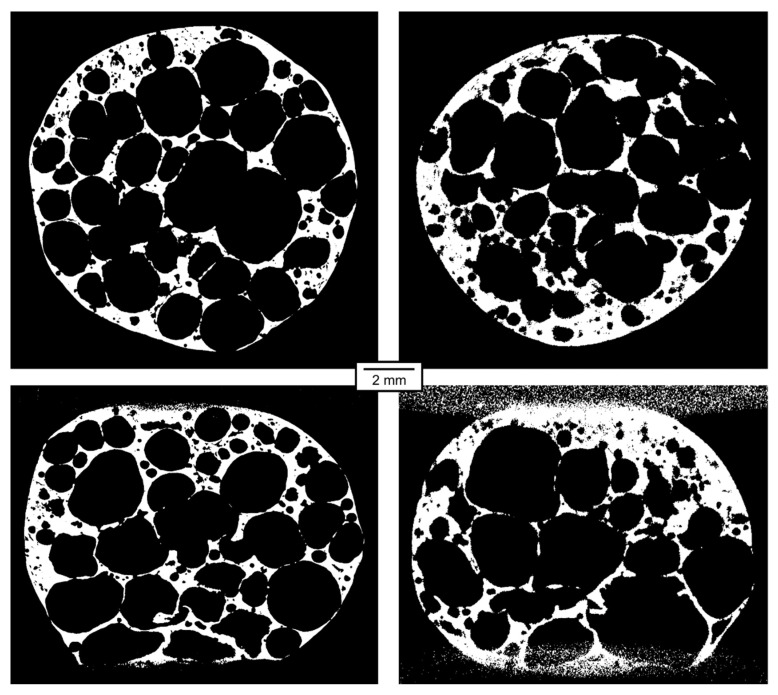
Comparison of the binarized section images from the 3D models reconstructed from the time-lapse tomography, Mode B, deformation 10% (**left**), and on-the-fly tomography, Mode E, deformation 11.4% (**right**).

Even with decreased CNR and increased FWHM, the results from the fast on-the-fly CT are usable for mechanical analyses. In [23], mechanical analyses were performed on tomographic models acquired exactly as in Mode B (800 projections per 360°, time-lapse CT). During the processing, the models used in [23] were binarized. Increased noise and lower resolution of the models acquired in Mode E and Mode F, although clearly visible, do not prevent the binarization of the models and their analyses identical to those described in [23]. The FWHM of approximately 100 µm allows a detection of the structural features of the same order. Figure 13 shows the comparison of binarized images from Mode B (time-lapse) and Mode E (on-the-fly). The binarization was made in the same way as in [23], where the binarized images were used for further mechanical analyses. It can be seen that even the on-the-fly results are usable for these analyses.

The time-lapse 4D tomographic experiments are very demanding and time-consuming. In this regard, the time for one CT scan is an important factor. The time-lapse tomography reported in this paper took 7 min for acquisition of 800 projections (Mode B). After that, the next loading step had to be performed and there was at least 2 min delay needed to avoid relaxation effects, before the next tomography could start. Therefore, one entire step of time-lapse tomography took approximately 10 min. On the other hand, the on-the-fly CT scan, although yielding lower image quality of the reconstructed model, could be done within 12 s. Due to the above-mentioned technical reasons (batch saving of the dataset), the achieved temporal resolution of the reported on-the-fly procedure was 24 s. Thus, 41 tomographies within 16 min were made. The comparable time-lapse tomography would take 410 min (almost 7 h) for the same task.

## 6. Conclusions

A microcomputed tomography experiment with a market available Dexela scintillation detector was performed using the exposure time of 15 ms, allowing the acquisition of a dataset of 800 projections per one revolution within 12 s, with the 2 px × 2 px binning and resolution of 972 px × 768 px. For technical reasons, every second revolution of the rotation platform was used to save the acquired images, so that 41 tomographies were acquired during 960 s (16 min). APM foam elements of approximately 10 mm in diameter were continuously deformed during the experiment, employing a table-top loading device. Two APM samples were tested, one with the total deformation of 24% and one with a faster loading and the total deformation of 48% during the same time. The resolution of the reconstructed volumes was 15.2 µm per voxel.

The results were compared to an experiment made in the time-lapse CT mode, i.e., with the platform suspended during the exposure and loading being suspended during the whole revolution. In this experiment, the exposure time was 90 ms and the resolution of the detector was 1944 px × 1536 px, giving a resolution of 7.6 µm per voxel in the reconstructed volumes. The difference in the image quality of the time-lapse and the on-the-fly experiments can be visually observed. The CNR of the time-lapse CT model was approximately 15, compared to approximately 8 in the case of the fast on-the-fly CT. The FWHM of line spread function was approximately 50 µm in the case of the time-lapse CT and approximately 70 µm in the case of the on-the-fly CT. In the experiment with faster loading, where the displacement during one rotation corresponded to approximately 4 px, the FWHM was approximately 100 µm in the direction of displacement. Hence, the image quality is undoubtedly lower in the case of the fast on-the-fly CT. However, if the resultant models are intended to be used for structural analyses which require binarization of the images, the results of the on-the-fly CT are still satisfactory.

As for the experiment duration, the time-lapse procedure of the same extend as the 16 min on-the-fly scanning would take around 7 h. Although the time for the experiment is an important factor, there is yet another aspect that should be addressed. During the on-the-fly experiment, one CT scan was made in 12 s, compared to 420 s in the time-lapse mode. Thus, the on-the-fly approach can be interesting in the cases, where it is desirable to keep the scanning time short. Such applications include scanning of the delicate biological samples, where it is important to avoid drying of the sample during scanning or ensure a low total radiation dose. When investigating the dynamical processes which are too fast to be scanned in a common way, where one scan takes units to tens of minutes, the on-the-fly approach also represents an interesting alternative.

## Figures and Tables

**Table 1:** Parameters of the six investigated acquisition modes.

Mode	CT Acquisition(TL = Time-Lapse, OTF = On-the-Fly)	Projections per 360° [–]	Angle change during exposure [°]	Tube target power [W]	Resolution w × h [px]	Detector acquisition mode (HW = High Well, LW = Low Well)	Exposure time [ms]	Duration of one CT scan [s]	Loading displace-ment per one rotation [µm]
**A**	TL	2400	0	30	1944 × 1536	HW	90	1260	0
**B**	TL	800	0	30	1944 × 1536	HW	90	420	0
**C**	OTF	2400	0.15	25	972 × 768	LW	15	36	0
**D**	OTF	800	0.45	25	972 × 768	LW	15	12	0
**E**	OTF	800	0.45	25	972 × 768	LW	15	12	30
**F**	OTF	800	0.45	25	972 × 768	LW	15	12	60

**Table 2:** CNR and FWHM of reconstructed tomographic volumes measured on transversal and lateral slices.

Mode	CNR transversal/% of best value	CNR lateral/% of best balue	FWHM transversal [µm]/% of best value	FWHM lateral [µm]/% of best value
**A**	18/100	18.1/100	47/100	52/104
**B**	14.3/79	15.6/86	49/104	50/100
**C**	14.5/81	13.5/75	71/151	72/144
**D**	8.5/47	10.7/59	85/181	70/140
**E**	7.7/43	8.6/48	74/157	74/148
**F**	8.7/48	10.7/59	70/149	100/200

## Data Availability

The data supporting the findings of this study are available from the corresponding author M.V. (Michal Vopalensky) on request.
